# Alpha7 nAChR Expression Is Correlated with Arthritis Development and Inhibited by Sinomenine in Adjuvant-Induced Arthritic Rats

**DOI:** 10.1155/2019/3759304

**Published:** 2019-05-02

**Authors:** Chong Peng, Qing-ping Shi, Jia-yan Liu, Yan-jun Lv, Jing Li, Lang Yi, Sha-sha Bai, Liang Liu, Pei-xun Wang, Hua Zhou, Ke-er Huang, Yan Dong

**Affiliations:** ^1^Department of Immunology, Institute of Clinical Pharmacology, Guangzhou University of Chinese Medicine, Guangzhou, China; ^2^The First Clinical Medical School, Guangzhou University of Chinese Medicine, Guangzhou, China; ^3^Faculty of Chinese Medicine and State Key Laboratory of Quality Research in Chinese Medicine, Macau University of Science and Technology, Avenida Wai Long, Taipa, Macau; ^4^International Institute of Translation Chinese Medicine, Guangzhou University of Chinese Medicine, Guangzhou, China

## Abstract

Sinomenine (SIN) is the active ingredient of the Chinese herb* Sinomenium acutum* that has been used to treat rheumatoid arthritis (RA) for about 30 years in China. Marked expression of the alpha7 nicotinic acetylcholine receptor (*α*7nAChR) in the joint synovium of RA patients suggested a relationship between *α*7nAChR and RA. This study investigated the relationship between *α*7nAChR and RA development and the effects of SIN on *α*7nAChR expression* in vivo* and* in vitro*. Sprague-Dawley rats were injected with complete Freund's adjuvant to induce arthritis and then treated with SIN or methotrexate (MTX) from day 0 to day 30. Four clinical parameters—paw volume, arthritic index (AI), serum TNF-*α* concentration, and erythrocyte sedimentation rate (ESR)—were measured. Splenic lymphocytes were isolated for Bacille Calmette Guerin (BCG) stimulation. *α*7nAChR expression in tissues and cells was examined by RT-PCR, western blot, immunofluorescence, flow cytometry, and immunohistochemistry. Cell proliferation was evaluated by the CCK-8 assay. The relationship between *α*7nAChR expression and the four clinical parameters was analyzed by single-factor correlation analysis. Our results showed that the paw volume, AI, TNF-*α* concentration, and ESR in adjuvant-induced arthritic (AIA) rats were reduced by SIN or MTX treatment. SIN decreased *α*7nAChR expression in tissues and cells compared to the model group, while MTX had no significant effect on *α*7nAChR expression. Moreover, there was a positive relationship between *α*7nAChR expression and paw swelling, AI, and TNF-*α* concentration. Splenic lymphocyte activation was accompanied by increased *α*7nAChR expression, while SIN treatment inhibited cell activation and downregulated *α*7nAChR expression. *α*7nAChR expression showed a positive correlation with the progression of RA in AIA rats that may involve lymphocyte activation. Different from MTX, the inhibition of SIN on *α*7nAChR expression might contribute to its antiarthritic effect, suggesting that SIN could be an important supplement to the treatment strategy for RA.

## 1. Introduction

Rheumatoid arthritis (RA) is a systemic inflammatory disease that affects 0.5-1.0% of the world population. RA is characterized by joint swelling, synovial inflammation, persistent synovial hyperplasia, cartilage and bone damage, and general disability. The severity and progression of RA are affected by susceptibility genes, environmental damage, epigenetic modifications, and posttranslational modifications [1, 2]. Recent studies found marked expression of the alpha7 nicotinic acetylcholine receptor (*α*7nAChR) in the synovial membranes of RA patients and RA fibroblast-like synoviocytes [3, 4]. *α*7nAChR is an important receptor in the cholinergic anti-inflammatory pathway (CAP) [5]. Treatment with *α*7nAChR agonists decreases proinflammatory factors level in experimental sepsis [6], neuroinflammation [7] and arthritis [5]. It was reported that a lack of *α*7nAChR increased the severity of collagen-induced arthritis (CIA) in *α*7nAChR^−/−^ mice [8]. On the other hand, another study found that a lack of *α*7nAChR inhibited development of arthritis [9]. At present, the role of *α*7nAChR in the progression of RA remains unclear.

Sinomenine (SIN; 7,8-didehydro-4-hydroxy-3,7-dimethoxy-17-methylmorphinane-6-one, C19H23NO4) is an active alkaloid derived from Sinomenium acutum (Family Menispermaceae), a Chinese herb used extensively in RA treatment in China [10, 11]. In a previous study, we demonstrated that lipopolysaccharide (LPS) stimulation elevated *α*7nAChR expression in macrophages, while SIN inhibited the inflammatory response and decreased *α*7nAChR expression [12].

In the present study, *α*7nAChR expression during different stages of RA was observed in an arthritis rat model to investigate the correlation between *α*7nAChR and the clinical progression of RA. In addition, the effects of SIN and methotrexate (MTX) on *α*7nAChR expression* in vivo* and* in vitro* were analyzed.

## 2. Materials and Methods

### 2.1. Animals

Male Sprague-Dawley rats (100-120 g) were housed in a climate-controlled environment (22-26°C at 40-70% humidity) with a 12-h light/dark cycle and given food and drink ad libitum. The animals were adapted to the housing conditions for 7 days prior to the experiments. All experiments were conducted in accordance with the National Institutes of Health guidelines approved by the Ethical Committee for Animal Experiments of Guangzhou University of Chinese Medicine. The rats were anesthetized with 3% sodium pentobarbital (1 mL/kg) and abdominal aortic blood was collected. Subsequently, the rats were sacrificed due to excessive blood loss.

### 2.2. Reagents and Drugs

Mineral oil was purchased from Bio-Rad (California, USA), SIN was purchased from Melonepharma (Dalian, China), and MTX was purchased from SPH Sine Pharmaceutical Laboratories Co., Ltd (Shanghai, China). M. Tuberculosis Des. H37 Ra and the ELISA kit for measuring TNF-*α* concentration were purchased from BD Biosciences (San Diego, California, USA). The monoclonal antibody against *α*7nAChR was purchased from Santa Cruz Biotechnology (Santa Cruz, California, USA), CHRNA7 rabbit polyclonal antibody was purchased from Proteintech (Chicago, USA), and DyLightTM488 fluorescence secondary antibody was purchased from eBioscience (California, USA). Bacille Calmette Guerin (BCG), a vaccine isolated from* Mycobacterium bovis*, was purchased from Chengdu Institute of Biological Products Co., Ltd (Sichuan, China).

### 2.3. Adjuvant-Induced Arthritis (AIA) Induction and Treatment

Complete Freund's adjuvant (CFA) was prepared according to established methods [13]. Rats were induced on day 0 by an injection of 0.1 mL of CFA (2.5 mg/mL) at the base of the tail through an intradermal route. After the CFA injection, AIA rats (n=8) were treated with SIN (120 mg/kg/d), MTX (7.8 mg/kg/w), or vehicle (PBS) orally from day 0 to day 30.

### 2.4. Clinical Evaluation of AIA Rats

Disease progression and severity were evaluated by measuring the arthritic index (AI) and paw volume of both hind paws on days 0, 6, 12, 18, 24, and 30 after CFA injection. There were 8 rats in each group at each time point. According to the degree of swelling and erythema around the paws, the paws were graded from 0 to 4; 16 was the maximum AI score for each animal [14]. The hind paw volume was measured in a volumetric chamber and represented as an average volume of both hind paws.

### 2.5. Pathology

On day 30 after CFA injection, the rats were sacrificed and the synovia removed. Specimens were fixed for 12 h in 4% paraformaldehyde, embedded in paraffin, cut into 4-*μ*m sections, and stained with hematoxylin and eosin (H&E).

### 2.6. Measurement of Proinflammation Cytokine Levels

Blood was collected, left at room temperature for 1 h, and then centrifuged at 3000 rpm for 10 min. The supernatant was collected, and TNF-*α* concentration was measured using a commercially available ELISA kit according to the manufacturer's instructions (BD Biosciences, San Diego, California, USA).

### 2.7. Measurement of Erythrocyte Sedimentation Rate(ESR)

Blood (120 *μ*L) was mixed with sodium citrate (30 *μ*L; 0.109 mol/L) and transferred into a 1.0-mm × 100-mm capillary (VWR International, West Chester, Pa., USA). The capillary was maintained at an angle of 45° for 15 min, and then the amount (in millimeters) of clear fluid present at the top portion of the capillary was recorded.

### 2.8. Immunofluorescence

Peritoneal macrophages were collected on day 30 after CFA injection [15], seeded in a 6-well plate (2×10^5^ cell/mL, 1 mL per well) and cultured for 4 h. Then the cells were washed, fixed in 4% paraformaldehyde, and washed again. Cells were treated with 1% Triton X-100 and washed. Nonspecific binding was blocked by 5% rabbit serum before the cells were incubated with the *α*7nAChR (1:200) antibody. Next, cells were incubated with the fluorescence secondary antibody in the dark. Finally, the cells were stained with DAPI and observed under the fluorescent microscope.

### 2.9. RT-PCR

Total mRNA was extracted from tissues and cells to obtain cDNA according to the manufacturer's protocol. The primers used and the product sizes were: *α*7nAChR (sense 5′- GGCCCGGAGAGGACAIAGG-3′; antisense 5′-CGGCCACATACGACCCCAGAGT-3′; product size 187 bp), IFN-*γ* (sense 5′-ATCTGGAGGAACTGGCAAAAGGACG-3′; antisense 5′-CCTTAGGCTAGATTCTGGTGACAGC-3′; product size 288 bp), IL-4 (sense 5′-GTTCTGCTTTCTCATATG-3′; antisense 5′-AGCGTGGACTCATTCACG-3′; product size 330 bp), and *β*-actin (sense 5′-CACCCTGTGCTGCTCACCGAGGCC-3′; antisense 5′- CCACACAGATGACTTGCGCTCAGG-3′; product size 720 bp). The PCR conditions were 95°C for 5 min, 40 cycles of 95°C for 15 s, 60°C for 30 s and 72°C for 30 min, and 72°C for 5 min. The PCR products were separated by 1.5% agarose gel electrophoresis and optical density was determined by the UVP gel analysis system (Quantity one, Bio-Rad).

### 2.10. Immunohistochemistry

Sections were deparaffinized and rehydrated with a series of gradient concentrations of alcohol and endogenous peroxidase activity was blocked with 3% hydrogen peroxide. Antigen repair was accomplished by microwaving in 0.01 M sodium citrate buffer solution and washing with PBS. Nonspecific binding was blocked with 5% BSA. Each section was then incubated with *α*7nAChR antibody (1:200) or PBS, followed by incubation with the anti-mouse HRP secondary antibody. Finally, the sections were treated with a diaminobenzidine substrate-chromogen solution and stained with hematoxylin.

### 2.11. Western Blot

Protein from tissues or cells was extracted for *α*7nAChR detection by western blot. For immunoblot analysis, 50 *μ*g of protein per lane was fractionated by SDS-PAGE and blotted onto a PVDF membrane (Bio-Rad, USA). The membrane was blocked by a 5% BSA solution for 2 h at room temperature, and then incubated with the *α*7nAChR antibody (1:200) and a GAPDH antibody (1:1000). After washing with TBST, the membrane was incubated with an anti-mouse IgG antibody (1:25,000). The membrane was washed with TBST, and bands were detected by the ECL detection method and exposed to X-ray film. The intensity of the bands was subsequently analyzed and quantified by ImageLab software.

### 2.12. Flow Cytometry

Spleens from control and AIA rats were obtained at day 24. Splenic lymphocytes were collected and incubated with CD3 (0.625 *μ*L/T) and B220 (1.25 *μ*L/T) antibodies, followed by incubation with the *α*7nAChR antibody (1:100). Subsequently, the cells were incubated with a secondary goat anti-rabbit fluorescent antibody. Finally, the cells were resuspended in 500 *μ*L of PBS and analyzed by flow cytometry.

### 2.13. Splenic Lymphocyte Assay

Splenic lymphocytes were incubated with SIN (400 *μ*m) or MTX (10 *μ*g/mL) in a 96-well plate, and 30 minutes later, BCG (5 *μ*g/mL) was added. After 72 h, cell proliferation was evaluated by the CCK8 assay, following the manufacturer's directions.

### 2.14. Statistical Analysis

Data were evaluated using one-way ANOVA with the Dunnett's multiple comparison post hoc test. Single-factor correlation analyses were used to analyze the relationship between *α*7nAChR expression and these four clinical parameters: paw volume, AI, serum TNF-*α* concentration, and ESR. Data were expressed as mean ± SD and P<0.05 was considered statistically significant. All data were analyzed by the SPSS 17.0 software.

## 3. Results

### 3.1. SIN Inhibits Clinical Progression in AIA Rats

**Figure 1:**
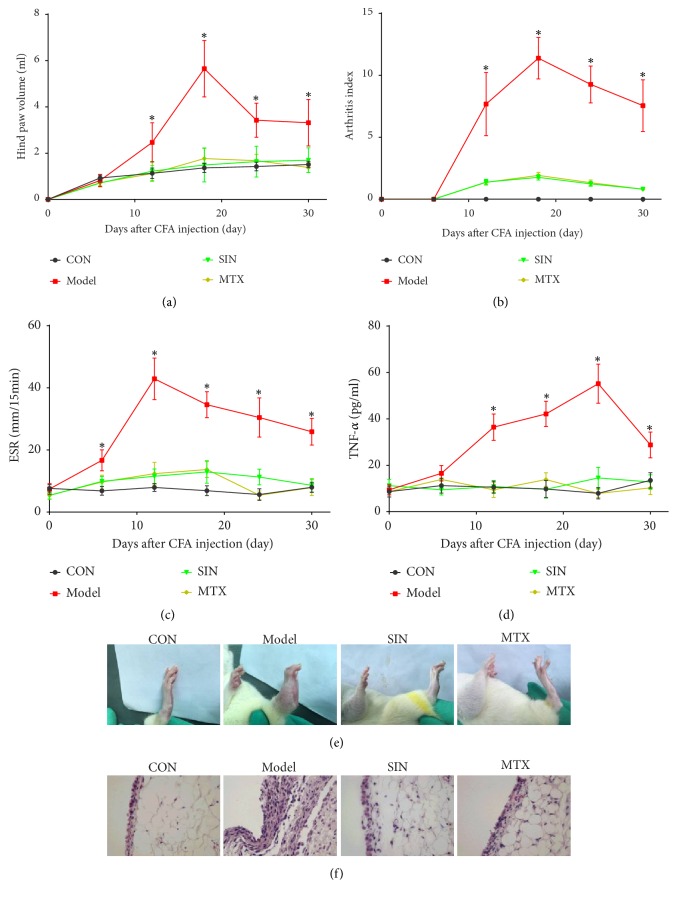
Effects of SIN on clinical progression in AIA rats from day 0 to 30. Rats were treated with SIN, MTX, or PBS once daily for 30 days after CFA administration. (a) Hind paw volume, (b) AI, (c) ESR, and (d) serum TNF-*α* concentration were measured on days 0, 6, 12, 18, 24, and 30. Data are expressed as mean ± SD (n=8). *∗*P<0.05 versus the model group. (e) Clinical appearance of hind paws from the control or AIA rats treated with SIN, MTX, or PBS, on day 30 after CFA administration. (f) Marked lining layer hyperplasia in the synovium of the model group compared to the control, SIN, or MTX group in H&E stained sections (400×).

Inflammatory arthritis is characterized by swelling and erythema in the paws. As shown in Figure 1, there was a significant increase in hind paw volume (Figure 1(a)), AI (Figure 1(b)), ESR (Figure 1(c)), and serum TNF-*α* concentration (Figure 1(d)) in the model group compared to the control group. Hind paw volume, AI, and TNF-*α* concentration increased from day 12 to 30, peaked on day 18 or 24, and then declined. ESR increased from day 6 to 30, peaked on day 12, and then declined. The above parameters were still higher than the control group on day 30. The clinical progression described above was inhibited by SIN or MTX. The hind paws of the rats were photographed and synovial tissues were isolated for assessment of histopathological changes on day 30. The model group showed severe soft tissue swelling and paw stiffness in comparison with the control group. In contrast, soft tissue swelling was significantly reduced by SIN or MTX (Figure 1(e)). Furthermore, the lining layer hyperplasia observed in the synovial tissues of the model group, but not the control group, was ameliorated by SIN or MTX (Figure 1(f)).

### 3.2. SIN Decreases *α*7nAChR Expression in Tissues and Peritoneal Macrophages of AIA Rats

**Figure 2:**
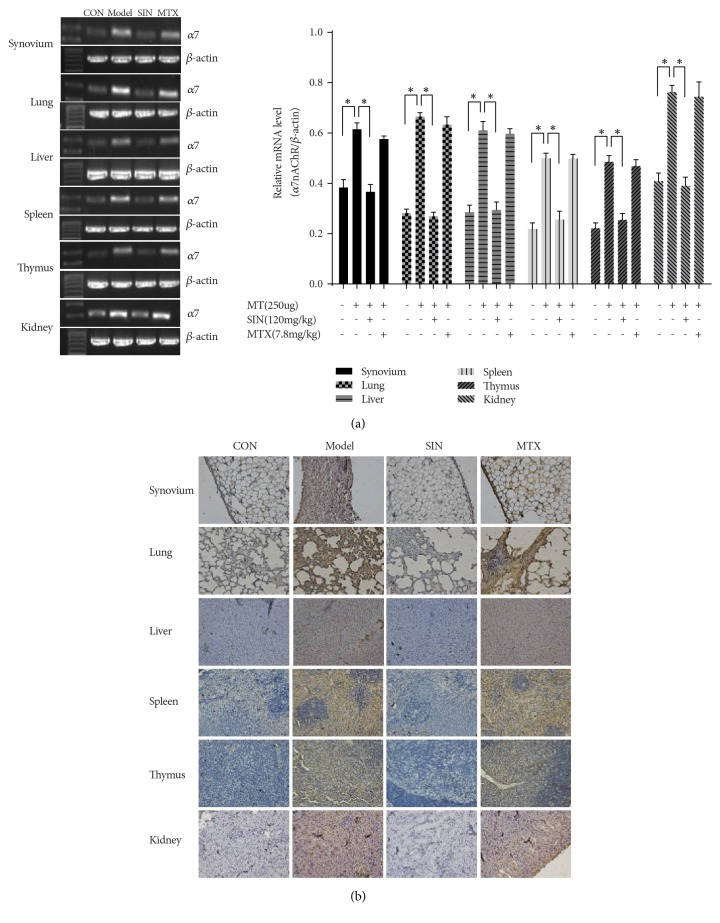
Effect of SIN on *α*7nAChR expression in AIA rats on day 30. Shown are representative results of (a) *α*7nAChR mRNA expression and (b) immunohistochemistry of *α*7nAChR in synovium, lung, liver, spleen, thymus, and kidney (200×). Data are expressed as mean ± SD (n=3). *∗*P<0.05 versus the model group.

**Figure 3:**
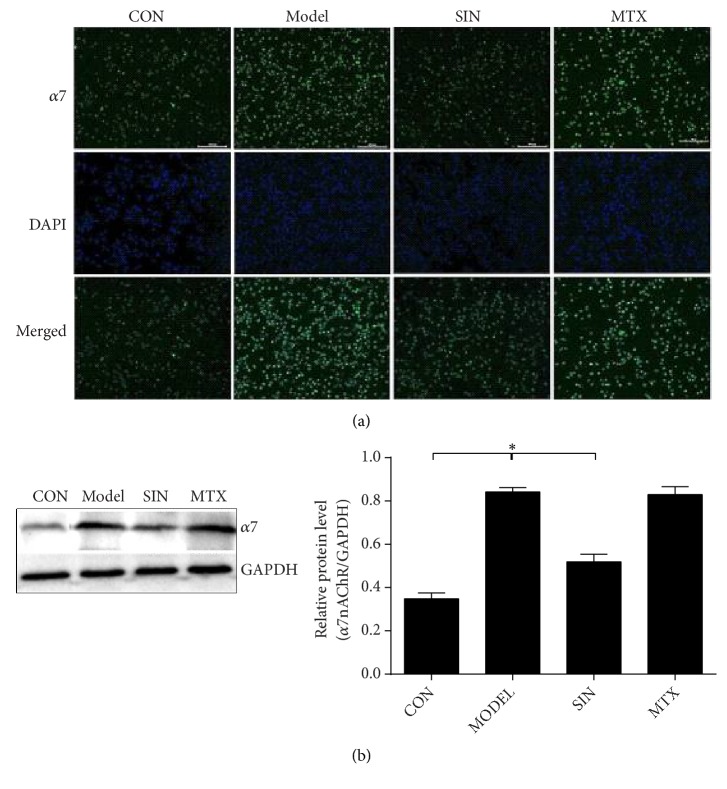
Effects of SIN on *α*7nAChR expression in peritoneal macrophages and MLN on day 30. (a) Immunofluorescence with antibodies specific for *α*7nAChR in peritoneal macrophages of control or AIA rats treated with SIN, MTX, or PBS (200×). (b) Protein expression of *α*7nAChR detected by western blot in MLN. Data are expressed as mean ± SEM (n=3). *∗*P<0.05 versus the model group.

The expression of *α*7nAChR in tissues, including the synovium, lung, liver, spleen, thymus, kidney, MLN, and peritoneal macrophages, was analyzed by RT-PCR, immunohistochemistry, immunofluorescence, and western blot. The results (Figures 2 and 3) showed that *α*7nAChR expression was elevated in the tissues of the model group compared with the control group. Treatment with SIN significantly decreased *α*7nAChR expression in all tissues evaluated when compared with the model group. In contrast, treatment with MTX did not have significant effects on *α*7nAChR expression in these tissues.

### 3.3. *α*7nAChR Expression Is Related to Clinical Progression and Regulated by SIN in AIA Rats

**Figure 4:**
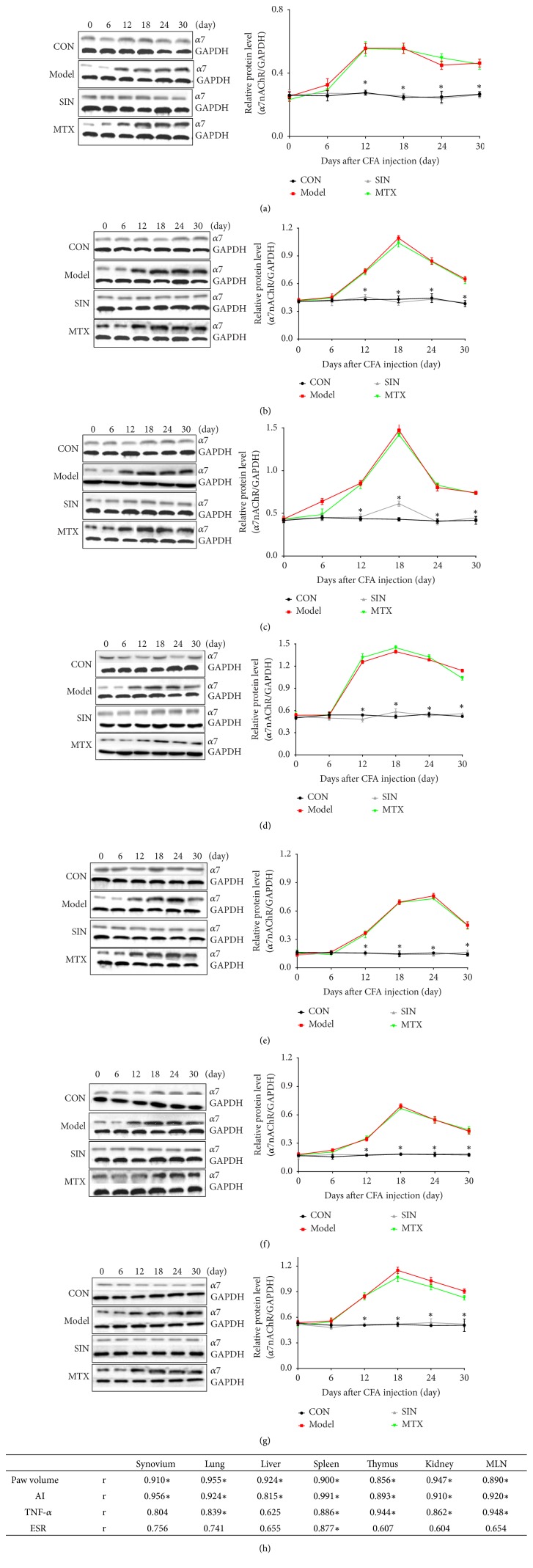
The time course of *α*7nAChR expression and correlation analysis with clinical progression in AIA rats. ((a)-(g)) The expression of *α*7nAChR protein detected by western blot in the synovium, lung, liver, spleen, thymus, kidney, and MLN of AIA rats on days 0, 6, 12, 18, 24, and 30. Data are expressed as mean ± SEM (n=3). *∗*P<0.05 versus the model group. (h) The correlation analysis between *α*7nAChR expression and clinical progression, including hind paw volume, AI, serum TNF-*α* concentration, and ESR. *∗*P<0.05 indicates statistical significance, r = 0.8-1.0 indicates very strong correlation, r = 0.6-0.8 indicates strong correlation, r = 0.4-0.6 indicates moderate correlation, r = 0.2-0.4 indicates weak correlation, and r = 0.0-0.2 indicates very weak or no correlation.

The protein levels of *α*7nAChR in various tissues were detected on days 0, 6, 12, 18, 24, and 30 by western blot, and the relationship between RA and *α*7nAChR was determined via a correlation analysis between *α*7nAChR expression and clinical progression. As shown in Figures 4(a)–4(g), *α*7nAChR expression in various tissues increased from day 12 to 30 and peaked on day 18 or 24 in the model group compared to the control group. Treatment with SIN markedly decreased *α*7nAChR expression from day 12 to 30 compared to the model group, while treatment with MTX had no significant effect on *α*7nAChR expression. The correlation analysis (Figure 4(h)) showed that hind paw volume and AI were strongly correlated with *α*7nAChR expression in the synovium, lung, liver, spleen, thymus, kidney, and MLN. Serum TNF-*α* concentration was strongly correlated with *α*7nAChR expression in the lung, spleen, thymus, kidney, and MLN, while ESR had no significant correlation with *α*7nAChR expression in any tissue.

### 3.4. *α*7nAChR Expression Is Related to the Activation of Lymphocytes and Regulated by SIN

**Figure 5:**
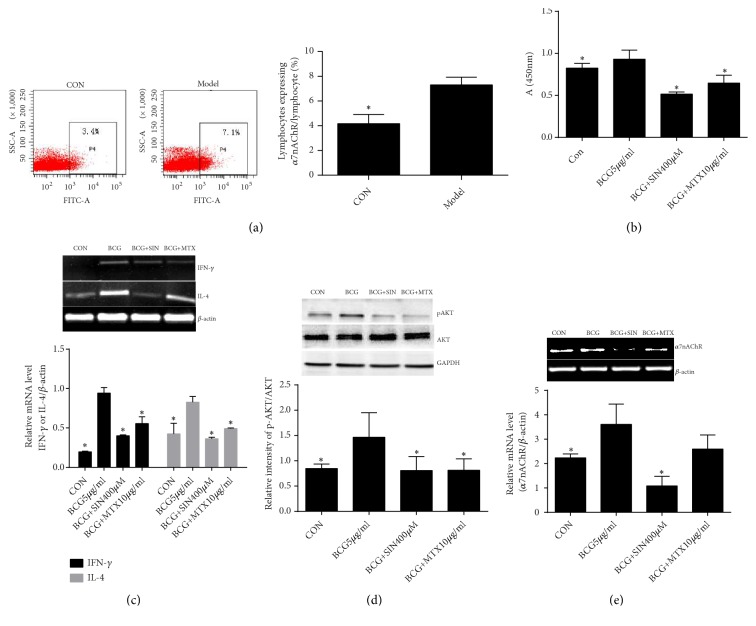
Effect of SIN on activated lymphocytes and *α*7nAChR expression. (a) Lymphocytes expressing *α*7nAChR in control or AIA rats were measured by flow cytometry (n=3). (b) Lymphocyte proliferation was detected by the CCK8 assay (n=5). mRNA expression of (c) IFN-*γ*, IL-4 and (e) *α*7nAChR was detected by RT-PCR and (d) protein expression of pAKT and AKT was detected by western blot in lymphocytes (n=3). Data are expressed as mean ± SEM. *∗*P<0.05 versus the model or BCG group.

As shown in Figure 5(a), *α*7nAChR expression was significantly increased in the splenic lymphocytes of AIA rats compared with control rats. We used BCG to stimulate the proliferation and activation of splenic lymphocytes from AIA rats* in vitro* to study the relationships between *α*7nAChR expression and lymphocyte activation. The proliferation of cells, expression of IFN-*γ* and IL-4, and protein level of pAKT were increased after BCG stimulation. However, SIN and MTX inhibited cell proliferation and decreased the expression of IFN-*γ*, IL-4, and the protein level of pAKT (Figures 5(b)–4(d)). Meanwhile, *α*7nAChR expression in splenic lymphocytes was increased after BCG stimulation, and *α*7nAChR expression was decreased by SIN (Figure 5(e)). However, MTX failed to decreased *α*7nAChR expression in activated splenic lymphocytes.

## 4. Discussion

*α*7nAChR has been identified as an essential player in the CAP, in which acetylcholine (ACh) released by the vagal nerve interacts with *α*7nAChR of nonneuronal cells such as macrophages to reduce excessive inflammation [16, 17]. Additionally, an anti-inflammatory effect of *α*7nAChR activated by agonists such as nicotine was verified in innate immunity cells such as macrophages and in mice stimulated by LPS [18–21]. Although it is well known that agonist-induced *α*7nAChR activation inhibits the production of inflammatory cytokines in macrophages, a few studies also provided evidence that the presence or expression of *α*7nAChR may be involved in pathological processes. For example, Guillermo reported that deletion of *α*7nAChR decreased lesion size, macrophage content, and cell proliferation in advanced lesions in mice, suggesting a proatherogenic role for *α*7nAChR in macrophages [22]. Inflammatory angiogenesis was markedly inhibited by inhibition of nAChR or deletion of *α*7nAChR [23]. In addition, a marked expression of *α*7nAChR was detected in the synovium of RA patients [3, 4].

In spite of these studies, the role of *α*7nAChR in RA remains largely unknown. In CIA mice, increased incidence and severity of arthritis was detected in *α*7nAChR^−/−^ mice compared to wild-type mice; this aggravation of arthritis might be due to the lack of *α*7nAChR and the resulting inability to respond to Ach [8]. On the contrary, a different study reported that a lack of *α*7nAChR inhibited disease development in CIA mice, implying that *α*7nAChR might be involved in adaptive immunity, and the alleviation of arthritis in *α*7nAChR^−/−^ mice might be related to a decreased proliferative immune response [9]. Another study showed that the severity and course of experimental autoimmune encephalomyelitis, a neuroinflammatory disease, was milder in *α*7nAChR^−/−^ mice than in wild-type mice. The lack of *α*7nAChR affects the reactivity of immune cells in a complex way, and one possible reason is that the deficiency of *α*7nAChR in antigen presenting cells reduces antigen presentation, implying a proinflammatory role for *α*7nAChR [24]. Moreover, increased expression of *α*7nAChR in human lymphocytes incubated with nicotine protected the lymphocytes from apoptosis [25]. Deletion of *α*7nAChR in B lymphocytes resulted in apoptosis and decreased immune response in mice [26, 27]. These previous studies suggest that the role of *α*7nAChR cannot be simply anti-inflammatory, especially in adaptive immunity cells and in autoimmune diseases; the results of this present study support this idea.

We studied the relationship between *α*7nAChR expression and the clinical progression of RA for the first time in AIA rats. We found an elevation of *α*7nAChR expression in tissues, peritoneal macrophages, and splenic lymphocytes of AIA rats, which was consistent with previous observations of *α*7nAChR expression in the synovium and fibroblast-like synoviocytes of RA patients [3, 4]. SIN exerted its anti-inflammatory action by ameliorating the clinical signs of RA, such as hind paw volume, AI, serum TNF-*α* concentration, ESR, and pathological changes of the synovium. In addition, SIN reduced *α*7nAChR expression in tissues and cells. On the other hand, MTX had no significant effect on *α*7nAChR expression, although it had a dramatic anti-inflammatory effect. Taken together, our results indicate that SIN plays an antiarthritic role accompanied with reduction of *α*7nAChR expression, which is different from the mechanism of action of MTX.

Furthermore, to investigate the relationship between *α*7nAChR expression and clinical progression of RA, we observed the time course of *α*7nAChR protein levels in various tissues of AIA rats on days 0, 6, 12, 18, 24, and 30. Our findings suggest that the pathogenesis and severity of RA are correlated with the elevation of *α*7nAChR expression. The correlation analysis between *α*7nAChR expression and the four clinical signs revealed a very strong correlation between hind paw swelling and AI and the *α*7nAChR level in the synovium, lung, liver, spleen, thymus, kidney, and MLN. A very strong correlation between serum TNF-*α* concentration and *α*7nAChR expression in lung, spleen, thymus, kidney, and MLN was also observed. These results suggest that *α*7nAChR might be involved in the occurrence and development of RA.

We isolated spleen lymphocytes to investigate whether *α*7nAChR is involved in lymphocyte activation in vitro. IFN-*γ* and IL-4 are produced by T cells and play an important role in their proliferation and differentiation [28]. Downstream target proteins during chemotherapy or radiotherapy such as caspase-9, Bad, and NF-*κ*B are regulated by activated AKT, which promotes cell proliferation and angiogenesis and inhibits apoptosis [29–32]. Phosphorylation of AKT can cause cell proliferation and antiapoptosis [33]. We found that, upon stimulation with BCG, the splenic lymphocytes of AIA rats proliferated significantly, and mRNA levels of IFN-*γ* and IL-4 and the protein level of p-AKT were increased. In addition, *α*7nAChR expression was increased in BCG-activated lymphocytes. While both SIN and MTX inhibited cell proliferation and phosphorylation of AKT and reduced IFN-*γ* and IL-4, only SIN downregulated *α*7nAChR expression. These data suggest that *α*7nAChR is involved in the activation of lymphocytes and is perhaps a target for SIN inhibiting the activation of lymphocytes.

## 5. Conclusions

In conclusion, the expression of *α*7nAChR increases when RA begins to develop, and *α*7nAChR has a positive correlation with the clinical progression of RA and lymphocyte activation in AIA rats. These findings indicate that *α*7nAChR may be a novel target for RA treatment. The antiarthritic effects of SIN were associated with diminished *α*7nAChR expression, whereas MTX had no significant impact on *α*7nAChR expression, indicating that the antiarthritic mechanism of SIN is different from MTX. Our results suggest that inhibition of *α*7nAChR expression by SIN might be an important supplement to the treatment strategy for RA. Further studies are required to elucidate the precise mechanisms underlying the involvement of *α*7nAChR in RA.

## Data Availability

The data used to support the findings of this study are available from the corresponding author upon request.

## Abbreviations

AI: Arthritic index
AIA: Adjuvant-induced arthritis
*α*7nAChR: Alpha7 nicotinic acetylcholine receptor
BCG: Bacille Calmette Guerin
CAP: Cholinergic anti-inflammatory pathway
CFA: Complete Freund's adjuvant
ESR: Erythrocyte sedimentation rate
MLN: Mesenteric lymph node
MTX: Methotrexate
RA: Rheumatoid arthritis
SIN: Sinomenine.
